# A novel adenine-based metal organic framework derived nitrogen-doped nanoporous carbon for flexible solid-state supercapacitor

**DOI:** 10.1098/rsos.171028

**Published:** 2018-01-31

**Authors:** Haowen Li, Dongying Fu, Xian-Ming Zhang

**Affiliations:** Institute of Crystalline Materials, Shanxi University, Taiyuan 030006, People's Republic of China

**Keywords:** metal organic framework, nitrogen-doped nanoporous carbon, electrochemical techniques, supercapacitor

## Abstract

In this article, we have synthesized a series of nitrogen-doped nanoporous carbon (NPC) from metal organic framework of UiO-66 with different ratios of adenine and 1,4-benzendicarboxylate (H_2_BDC) coated on carbon nanotube film (CNTF) to obtain a flexible porous electrode (NPC/CNTF). It is worth noting that the introduction of adenine at different ratios did not change the structure of UiO-66. We also investigated the effect of carbonization temperature from 800 to 1000°C on the electrochemical properties of the NPC. The ratio (H_2_BDC:adenine) 9 : 1 and the NPC carbonized at 900°C (denoted as NPC-1-900) exhibits better electrochemical properties. The results show that NPC-1-900/CNTF electrode exhibits an exceptional areal capacitance of 121.5 mF cm^−2^ compared to that of PC-900/CNTF electrode (22.8 mF cm^−2^) at 5 mV s^−1^ in a three-electrode system, indicating that the incorporation of nitrogen is beneficial to the electrochemical properties of nanoporous carbon. A symmetric flexible solid-state supercapacitor of NPC-1-900/CNTF has also been assembled and tested. Electrochemical data show that the device exhibited superior areal capacitance (43.2 mF cm^−2^) at the scan rate of 5 mV s^−1^; the volumetric energy density is 57.3 µWh cm^−3^ and the volumetric power density is 2.4 mW cm^−3^ at the current density of 0.5 mA cm^−2^ based on poly(vinyl alcohol)/H_3_PO_4_ gel electrolyte. For practical application, we have also studied the bending tests of the device, which show that the device exhibits outstanding mechanical stability under different bending angles. Furthermore, the flexible device shows excellent cyclic stability, which can retain 91.5% of the initial capacitance after 2000 cycles.

## 1. Introduction

During the last few decades, due to the increasingly serious energy shortages and environmental pollution, development of high-efficiency and environmentally friendly energy storage devices with high power output and long life cycles has attracted attention [1,2]. Compared with other energy storage devices, supercapacitors (SCs) have higher power density, excellent cycle stability and high charge–discharge efficiency [3–5]. Especially, with the increasing demand for wearable and foldable electronic products, flexible solid-state supercapacitors (SSCs) have increasingly evolved to meet the large proliferation of consumer electronics [6,7]. However, there are still some drawbacks, such as low energy density and high cost, which limits their practical applications [8]. To circumvent these shortcomings, it is necessary to develop new electrode materials in order to enhance efficiency and practicability [9–11].

Owing to the excellent physical and chemical stability, low cost and available, high specific surface area and good conductivity, porous carbon (PC) materials, such as activated carbon [12], nanoporous carbon [13], carbon nanotubes [14] and graphene [15], are widely used as electrode materials in SCs [16]. Wang *et al*. showed enhanced electrochemical properties through the synthesis of all-carbon layer-by-layer motif architectures by introducing two-dimensional ordered mesoporous carbons within the interlayer space of two-dimensional nanomaterials [17]. However, pure carbon materials exhibit poor hydrophilicity, which reduced the electrochemical performance of electrode materials [18]. In terms of enhancing the hydrophilicity of PC materials, nitrogen-doped porous carbons (NPC) are considered the most promising because the doped N atoms can provide a pair of electrons, which modify hydrophilicity and wettability of PC materials. Moreover, the doped N atoms can enhance the conductivity and capacitance of PC materials [19,20]. NPC materials have been prepared through a variety of methods, such as pyrolysis of organic precursors and carbonization of nitrogen-containing precursors [21–24]. However, the synthesis methods mentioned above are complex, expensive and the pore size distribution of carbon materials is disordered. It is well known that electrochemical performances of carbon materials are generally decided by their electrical conductivity, pore structures and specific surface area [25]. So, it is necessary to develop new methods and new materials, which can yield carbon materials with high surface area and uniform pore structures.

Lately, metal organic frameworks (MOFs) have been used for electrode materials of SCs due to their tremendous surface area, permanent porosity and controllable structures [26–28]. The surface area of MOFs ranges from 1000 to 10 000 m^2^ g^−1^, and pore size can be modified as large as 9.8 nm [26]. However, owing to the low conductivity of pristine MOFs, their application is limited in the electrochemical field. Recently, literature reports a large number of methods to improve their electrochemical capability. For example, Saraf *et al*. designed a novel Cu-MOF/rGO hybrid as supercapacitor electrode material and electrochemical nitrite sensor [29]. It is worth noting that MOFs are outstanding precursors and templates for the preparation of PC materials with high porosity, particularly for hierarchical nanostructures [30,31]. Compared with traditional method, MOF-derived PC materials show some distinct advantages in terms of simple preparation and precise control over porous structure. Up to now, there are lots of research developments on the preparation of MOF-derived PC materials for supercapacitor application [30–32]. For the first time, Xu *et al*. demonstrated the application of MOF-5 as a sacrificial template for synthesis of porous carbons; the surface area of porous carbon is 2872 m^2^ g^−1^ and the specific capacitance is 258 F g^−1^ at the current density of 250 mA g^−1^ [33]. Moreover, due to the abundant nitrogen in the organic ligands, NPC materials can be easily synthesized through direct pyrolysis of nitrogen-containing MOFs. Nune *et al*. used IRMOF-3 as self-sacrificial template to prepare NPC materials; comparing with the nitrogen-free PC materials (the specific capacitance is 24 F g^−1^), the surface area of NPC is 553 m^2^ g^−1^, and the specific capacitance is 239 F g^−1^ at 5 mV s^−1^ [34], which indicated the importance of N-doping in carbon materials.

In general, NPC materials are prepared through pyrolysis of nitrogen-containing MOFs, such as zeolitic imidazolate frameworks [35,36]. It is necessary to introduce nitrogen source for MOFs without nitrogen. In this work, we provide a new method for preparing NPC materials, in which we added nitrogen ligand (adenine) to UiO-66 by adjusting the content of 1,4-benzendicarboxylate (H_2_BDC) and adenine and then prepared NPC at different calcination temperature. It is worth noting that the introduction of adenine at different ratios did not change the structure of UiO-66. The ratio (H_2_BDC : adenine) 9 : 1 and the NPC carbonized at 900°C (denoted as NPC-1-900) exhibits better electrochemical properties. The NPC-1-900/carbon nanotube film (CNTF) electrode material shows a maximum areal capacitance of 121.5 mF cm^−2^ which is higher than that of PC-900/CNTF electrode (22.8 mF cm^−2^) at 5 mV s^−1^ with a three-electrode system, indicating that the N-doping improves the capacitive performance of porous carbon. Compared with counterparts [25], the performance of NPC-1-900/CNT electrode material is excellent. For the needs of practical application, a symmetric flexible SSC has also been assembled and tested.

## 2. Experimental methods

### 2.1. Materials

The chemical reagents used in the experiments were as follows. CNTF (Hengqiu Tech. Inc., Suzhou), zirconium chloride (ZrCl_4_, 99.5%), 1,4-benzendicarboxylate (H_2_BDC, 98%), adenine (C_5_H_5_N_5_, 98%), the reagents mentioned above being purchased from Energy Chemical. *N*,*N*-dimethylformamide (HCON(CH_3_)_2_, DMF, 99.8%), ethanol (CH_3_CH_2_OH, 99.7%), methanol (CH_3_OH, 99.5%), sulfuric acid (H_2_SO_4_, 95.0--98.0%), phosphoric acid (H_3_PO_4_, 85%) and acetic acid glacial (CH_3_COOH, 99.5%) were purchased from Tianjin Chemical Works. Other reagents were poly(vinyl alcohol) (PVA-124, MW 105000, 99%, hydrolysed), 1-methyl-2-pyrrolidinone (NMP, 99.0%, Aladdin), poly(vinylidenefluoride) (PVDF, Sigma-Aldrich). Prior to use, the CNTF was ultrasonically cleaned in ethanol and deionized water successively. Deionized water was used throughout all the experiments. All the reagents and materials were of analytical grades and were used as received without further purification.

### 2.2. Synthesis of UiO-66 and adenine-UiO-66

UiO-66 was prepared by mixing ZrCl_4_ (0.7 g, 3 mmol) and H_2_BDC (0.5 g, 3 mmol) in 10 ml DMF under vigorous stirring for about 30 min at room temperature; 0.5 ml CH_3_COOH was then added with stirring. The obtained mixture was sealed and heated at 120°C for 24 h. After cooling naturally, the as-synthesized solid was rinsed by DMF and centrifuged three times, and then the product was purified with anhydrous methanol to exchange the guest DMF molecules for 3 days. After that, the UiO-66 was activated at 110°C for 24 h.

A series of adenine-UiO-66 with different content of adenine were prepared by adjusting the mole ratio of H_2_BDC and adenine (9 : 1, 8 : 2, 7 : 3, 6 : 4, 5 : 5), using the same procedure as in the preparation of UiO-66. Correspondingly, the as-prepared samples are denoted as U-X, where X is the different mole ratio of H_2_BDC and adenine, such as 1–5.

### 2.3. Synthesis of N-doped porous carbon

After excluding air by flowing Ar gas for 3 h, the as-synthesized UiO-66 and U-X were heated at 500°C for 3 h at a heating rate of 5°C min^−1^ under an Ar flow. The products were further carbonized at different temperature (800–1000°C) for 5 h and then immersed into 20% HF aqueous solution to yield the carbon materials. After washing with deionized water several times and drying overnight at 80°C, a series of PC and NPC-X were obtained. Correspondingly, the as-prepared samples are referred to as PC-(800, 900, 1000) and NPC-X-(800, 900, 1000).

### 2.4. Fabrication of PC/CNTF and NPC-X/CNTF electrodes

The fabrication of PC/CNTF and NPC-X/CNTF electrode materials was carried out as follows: the slurry of 80 wt % active material (PC or NPC-X) and 20 wt % PVDF binder in NMP was coated on CNTF. The mass of the active material coated on the film was about 1 mg for each electrode. The CNTF with 1 mg active materials was dried at 80°C for 12 h as the working electrode. The CNTF used for electrochemical measurement was conductive on one side, and the other side was non-conductive with insulating tape, 1.0 cm × 0.5 cm conductive areas as the substrate.

### 2.5. Fabrication of flexible symmetric solid-state supercapacitor device

The preparation of polymer-gelled electrolyte was described in our previous work [37]. For the fabrication of the device, two identical pieces of NPC-1-900/CNTF electrode with an area of 1.0 × 0.5 cm^2^ were placed in parallel and PVA/H_3_PO_4_ was used as the polymer-gelled electrolyte. The average thickness of the electrolyte separator is about 0.3 mm in the device and it acts as both the electrolyte and the ion-porous separator. The assembled device is placed at room temperature for 2 h, so that the electrolyte could completely penetrate the electrode.

### 2.6. Morphological and structural characterizations

Thermogravimetric analysis (SetaramLabsys Evo) was conducted up to 1000°C at a heating rate of 5°C min^−1^ under nitrogen atmosphere to evaluate the thermal behaviour of the samples. The morphologies of the samples were observed by using a JEOL-JSM-6701 field-emission scanning microscope (SEM) operating at an accelerating voltage of 10 kV and an FEI Tecnai G2 F20S-Twin transmission electron microscope (TEM) using an accelerating voltage of 200 kV. Powder X-ray diffraction (PXRD) measurements were recorded with a Rigaku Ultima IV diffractometer using Cu K*α* radiation and graphite monochromator (*λ* = 1.54056 A) at 40 kV voltage and 40 mA current at the scan speed of 5° min^−1^ with a step size of 0.02°. The N_2_ adsorption–desorption isotherms were obtained with a Quantachrome Autosorb iQ instrument; a liquid nitrogen bath (77 K) and ultra-high purity grade nitrogen and helium were used for the nitrogen adsorption experiment. All samples were degassed under vacuum at 393 K for 6 h before test. For calculation of the apparent surface areas, the multipoint Brunauer–Emmett–Teller (BET) method was applied using the adsorption branches of the N_2_ isotherms and the pore size distribution was analysed by density functional theory (DFT) and Barrett Joyner Halenda. X-ray photoelectron spectroscopy (XPS) was conducted with a PHI-5702 instrument. The atomic content of nitrogen was measured by Varion EL CUBE elemental analyser. Before measurements, all the samples were evacuated and activated at 120°C for 24 h under vacuum to remove any residual solvent.

### 2.7. Electrochemical properties measurements

Cyclic voltammetry (CV), electrochemical impedance spectroscopy (EIS) and galvanostatic charge/discharge (GCD) of CNTF, PC/CNTF and NPC-X/CNTF electrodes were evaluated from an electrochemical workstation SI 1287 and SI 1260 (Solaitron, England) using a three-electrode cell in 1 M H_2_SO_4_ electrolyte, with silver chloride electrode and Pt sheet as the reference electrode and counter electrode, respectively. The performance of the assembled device was tested by CV, GCD and EIS in a two-electrode configuration. The EIS data were studied in a frequency range of 10–100 mHz at an open circuit potential of 5 mV. The GCD test of all electrode materials and device was conducted at varying current density with the cutoff voltage of 0 and 1.0 V. All tests were carried out at room temperature. The calculation of the capacitance (*C*), power density (*P*) and energy density (*E*) was carried out as described in our previous paper [37].

## 3. Results and discussion

### 3.1. The characterization of the electrode material

#### 3.1.1. Thermogravimetric analysis

In order to analyse the thermal stability of as-synthesized UiO-66 and U-1, thermogravimetric analysis was conducted and shown in electronic supplementary material, figure S1. From the electronic supplementary material, figure S1, we can see that the curves of UiO-66 and U-1 were very similar indicating that the incorporation of adenine did not change the stability of UiO-66. At the beginning, the physically adsorbed H_2_O, guest H_2_O and DMF solvent molecules of the UiO-66 and U-1 were removed by heating from 30°C to 200°C. On further heating, a weight loss between 500°C and 600°C may be ascribed to the collapse of the structure, which was similar to the previous reported researches [38]. It is worth noting that the weight loss of UiO-66 and U-1 was 60 and 55 wt%, respectively, which might be ascribed to changes in the chemical environment due to the introduction of the adenine.

#### 3.1.2. Powder X-ray diffraction

**Figure 1.**
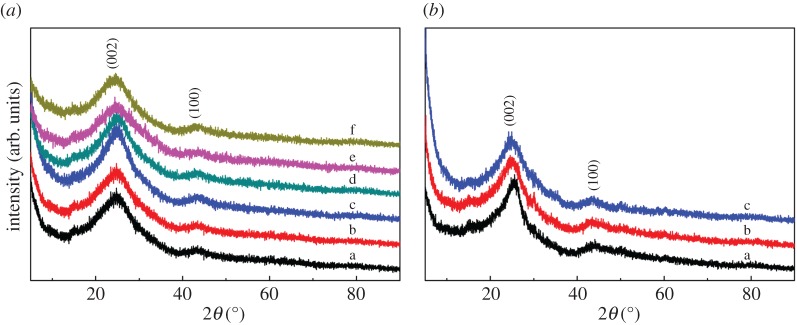
(*a*) Powder X-ray diffraction patterns of PC-900 (a), NPC-1-900 (b), NPC-2-900 (c), NPC-3-900 (d), NPC-4-900 (e) and NPC-5-900 (f). (*b*) Powder X-ray diffraction patterns of simulated NPC-1-800 (a), NPC-1-900 (b), NPC-1-1000 (c).

PXRD has been carried out to analyse the composition of all samples. As shown in the electronic supplementary material, figure S2, in comparison with the simulated PXRD pattern of UiO-66 [39], the as-synthesized UiO-66 and U-X showed a similar structure, which indicated that the introduction of adenine did not change the structure of UiO-66. The reason for this phenomenon may be that the adenine occupied the void spaces of UiO-66. As seen from figure 1*a*,*b*, two obvious broad peaks in all samples are observed at around 25° and 44°, which are corresponding to the (002) and (100) planes of graphitic carbon, respectively [40,41].

#### 3.1.3. Nitrogen adsorption measurements

**Figure 2.**
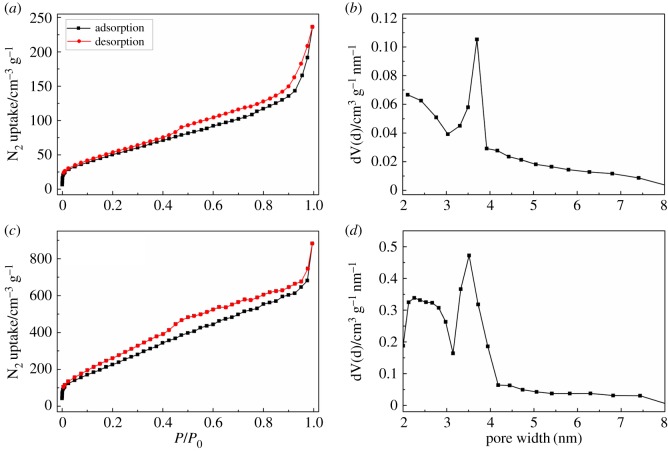
N_2_ adsorption/desorption isotherms of PC-900 (*a*); the pore size distribution of PC-900 (*b*); N_2_ adsorption/desorption isotherms of NPC-1-900 (*c*); the pore size distribution of NPC-1-900 (*d*).

The surface area and porosity of samples were investigated by nitrogen adsorption/desorption measurement. The results of UiO-66 and U-1 are shown in the electronic supplementary material, figure S3. It is worth noting that the BET specific surface area of U-1 is 1375 m^2^ g^−1^ (electronic supplementary material, figure S3C), which is larger than that of UiO-66 (1193 m^2^ g^−1^) (electronic supplementary material, figure S3A). Both UiO-66 and N-U-1 exhibited a type I isotherm. Moreover, the pore size distribution calculated by DFT model from the nitrogen desorption isotherm is shown in the electronic supplementary material, figure S3B,D. The average pore diameter of UiO-66 and N-U-1 is 0.785 nm, which is in the range of the values reported in [42]. The nitrogen adsorption/desorption isotherms of PC-900 and NPC-1-900 are shown in figure 2. Both PC-900 and NPC-1-900 exhibited a type IV isotherm. It is evident from figure 2 that the specific surface area of NPC-1-900 (973 m^2^ g^−1^) is larger than that of PC-900 (200 m^2^ g^−1^). The average pore diameter of U-1 is 3.514 nm, which is smaller than that of PC-900 (3.702 nm). The high specific surface area of NPC-1-900 may be due to the introduction of adenine molecules in the pores of UiO-66. Moreover, porous carbon with mesoporous structure is beneficial to improve the performance of SCs.

#### 3.1.4. Morphology characterization

**Figure 3.**
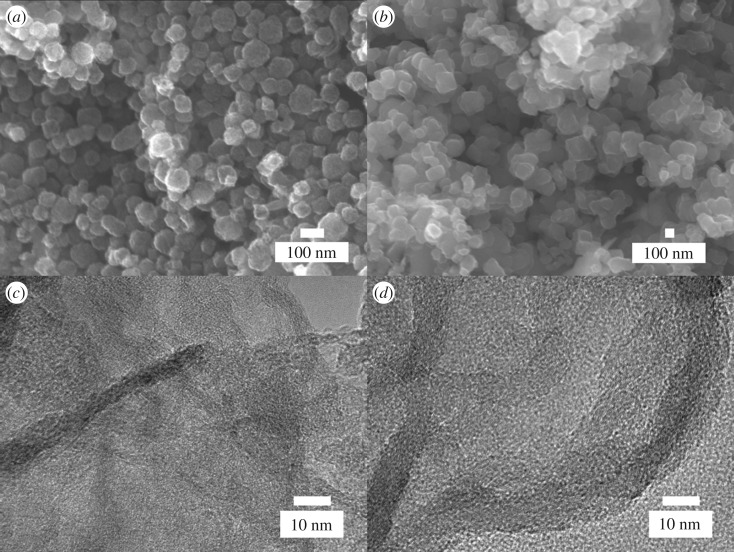
SEM images of PC-900 (*a*) and NPC-1-900 (*b*). TEM images of PC-900 (*c*) and NPC-1-900 (*d*).

The morphology and microstructure of all the samples were observed by SEM and TEM. Electronic supplementary material, figure S4A and figure S4B show the SEM images of UiO-66 and U-1, respectively. Both UiO-66 and U-1 were of cubic shape. It was further proved that the structure of UiO-66 did not change after the introduction of adenine molecules. The SEM images of PC-900 and NPC-1-900 are shown in figure 3*a*,*b*; it can be observed that these particles have good uniformity after calcination. The obtained porous carbon still maintained the structure of UiO-66. Moreover, SEM images revealed that the surface of porous carbon samples became rough after carbonization and HF washing. The TEM images of PC-900 (figure 3*c*) and NPC-1-900 (figure 3*d*) show that the amorphous carbon possessed lots of pores over the entire particle surface.

#### 3.1.5. X-ray photoelectron spectroscopy and elemental analysis

**Figure 4.**
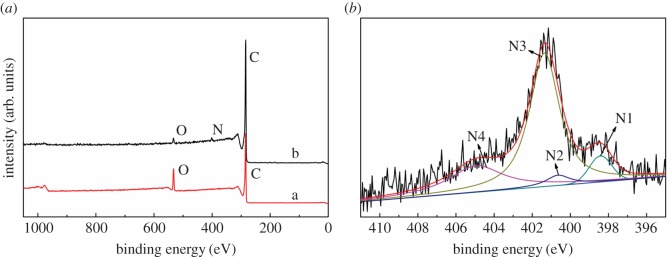
(*a*) XPS spectra of samples PC-900 (a) and NPC-1-900 (b). (*b*) High resolution N1s XPS spectrum of NPC-1-900.

It is necessary to clarify whether the nitrogen is introduced into the porous carbon surface by XPS. XPS of PC-900 (figure 4*a*-a) revealed two binding peaks at 533.08 eV and 286.08 eV, which were assigned to O1 s and C1s, respectively. In comparison with the curves of PC-900, the NPC-1-900 (figure 4*a*-b) shows another binding peak at 401.08 eV, which was assigned to N1 s. It shows that the NPC was successfully prepared by the introduction of adenine molecules. Moreover, the chemical state of the nitrogen on the surface of NPC-1-900 was further studied by the high-resolution N1 s region XPS analysis. From the XPS spectra of NPC-1-900 corresponding to N1 s transition in figure 4*b*, the signals of pyridinic-N (N1, 398.2 ± 0.2 eV), pyrrolic/pyridine-N (N2, 399.8 ± 0.2 eV), graphitic-N (N3, 401.6 ± 0.2 eV) and terminal N-O (N4, 404.06 ± 0.2 eV) bonding are shown by means of XPS deconvolution [43–45]. The result further confirmed the nitrogen doping of the porous carbon. The nitrogen atomic content of NPC-1-900 is 2.61% by elemental analysis. This indicated that we successfully obtained NPC by carbonized adenine-based MOF.

### 3.2. Electrochemical properties

**Figure 5.**
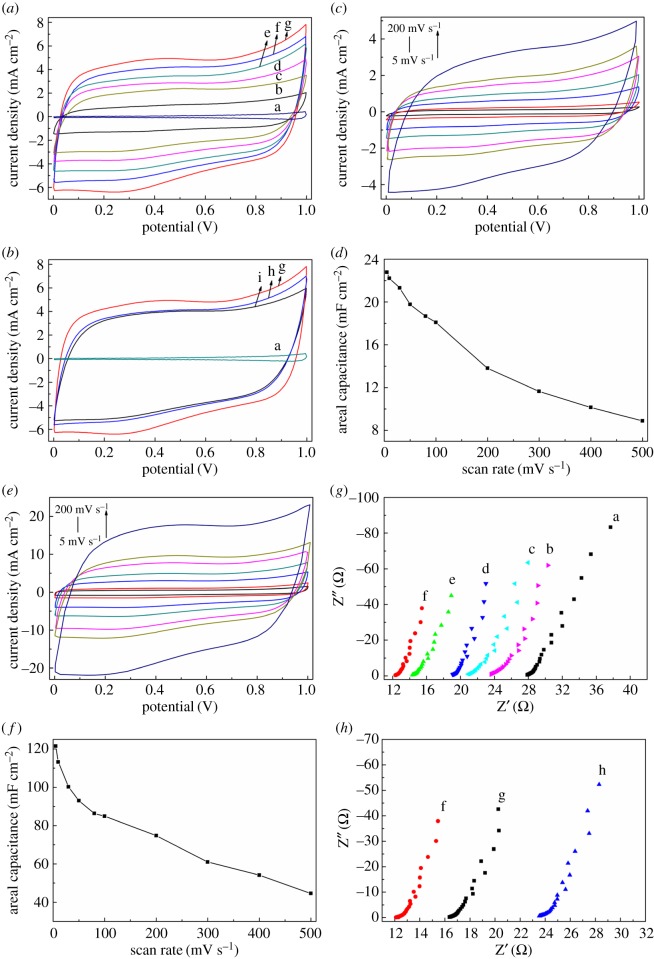
CV behaviours of CNTF, PC-900/CNTF and NPC-X-900 with different ratio (1, 4-benzendicarboxylate: adenine) (*a*) and NPC-1 at different calcination temperature (*b*) of CNTF (a), PC-900/CNTF (b), NPC-5-900/CNTF (c), NPC-4-900/CNTF (d), NPC-3-900/CNTF (e), NPC-2-900/CNTF (f), NPC-1-900/CNTF (g), NPC-1-800/CNTF (h), NPC-1-1000/CNTF (i) at scan rate of 50 mV s^−1^. CV behaviours of PC-900/CNTF (*c*) and NPC-1-900/CNTF (*e*) at scan rate of 5--200 mV s^−1^ (from bottom to top is 5, 10, 30, 50, 80, 100 and 200 mV s^−1^). Areal capacitance relationship of PC-900/CNTF (*d*) and NPC-1-900/CNTF (*f*) at different CV scan rates. Nyquist electrochemical impedance spectra (*g,h*) of PC-900/CNTF (a), NPC-5-900/CNTF (b), NPC-4-900/CNTF (c), NPC-3-900/CNTF (d), NPC-2-900/CNTF (e), NPC-1-900/CNTF (f), NPC-1-800/CNTF (g), NPC-1-1000/CNTF (h).

**Figure 6.**
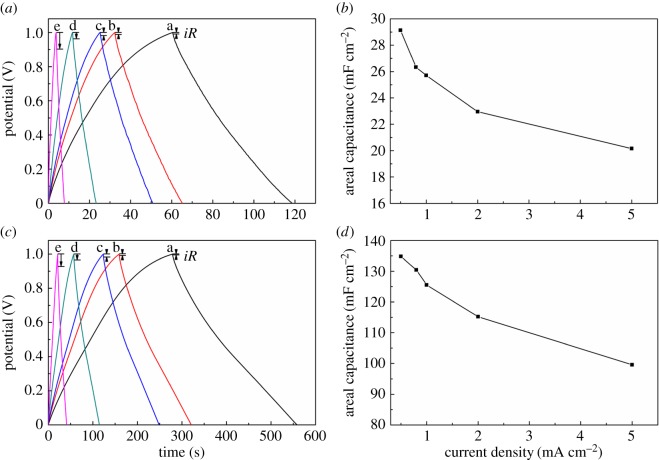
GCD curves of the PC-900/CNTF (*a*) and NPC-1-900/CNTF (c) electrodes at different GCD current densities (a 0.5, b 0.8, c 1, d 2 and e 5 mA cm^−2^). Areal capacitance relationship of the PC-900/CNTF (*b*) and NPC-1-900/CNTF (*d*) electrodes with different current densities.

The areal capacitance of electrode materials is of greatest importance, especially in flexible and wearable electronics applications [6,46]. The effects of different ratio (H_2_BDC: adenine) and calcination temperature on the properties of electrode materials are shown in figure 5*a*,*b*. As can be seen from figure 5*a*,*b*, NPC-1-900/CNTF electrode shows a larger areal capacitance than other electrodes at 50 mV s^−1^. In order to further investigate the performance of the samples, the CV curves of PC-900/CNTF (figure 5*c* and electronic supplementary material, figure S5A) and NPC-1-900/CNTF (figure 5*e* and electronic supplementary material, figure S5B) electrode materials were carried out at different scan rates from 5 to 500 mV s^−1^. As shown in figure 5*c* (electronic supplementary material, figure S5A) and figure 5*e* (electronic supplementary material, figure S5B), the CV curves show quasi-rectangular shapes from 5 to 500 mV s^−1^, which indicate a good rate behaviour. The plots of areal capacitance versus CV scan rates for PC-900/CNTF (figure 5*d*) and NPC-1-900/CNTF (figure 5*f*) show that the NPC-1-900/CNTF electrode displays an areal capacitance of 121.5 mF cm^−2^ while it is 22.8 mF cm^−2^ for PC-900/CNTF at 5 mV s^−1^. The electrochemical impedance diagrams of all samples are shown in figure 5*g*,*h*. Figure 5*g* shows the Nyquist plots of PC-900 (a) and NPC-X-900 (b--f) (X: 5, 4, 3, 2, 1, respectively) and figure 5*h* shows is the Nyquist plots of NPC-1 (f--h, 900°C, 800°C, 1000°C, respectively). The data show that the NPC-1-900/CNTF exhibits a lower charge transfer resistance than other electrodes, which verify the better electronic properties of NPC-1-900/CNTF. On the other hand, the GCD curves of PC-900/CNTF and NPC-1-900/CNTF electrodes were obtained at various current densities from 0.5 to 5.0 mA cm^−2^ in figure 6*a*,*c*, respectively. We can see that the GCD curves of both PC-900/CNTF and NPC-1-900/CNTF electrodes exhibit typical symmetric triangular shape, which indicated the electric double layer energy storage mode and an excellent fast charge–discharge performance. However, the performance of NPC-1-900/CNTF electrode is obviously better than that of PC-900/CNTF electrode. Based on the GCD curves of PC-900/CNTF and NPC-1-900/CNTF electrodes at the current density of 0.5 mA cm^−2^, the areal capacitance was 29.1 mF cm^−2^ and 134 mF cm^−2^, respectively. It is worth noting that the internal resistance loss (iR drop) observed from the discharge curve is small even at high current density, indicating a low internal resistance of the NPC-1-900/CNTF electrode material. Figure 6*b*,*d* shows the plots of the areal capacitance versus current density for the PC-900/CNTF and NPC-1-900/CNTF electrodes. The highest areal capacitance of 134 mF cm^−2^ for NPC-1-900/CNTF electrode can be obtained at a current density of 0.5 mA cm^−2^ with good retention of 77.8% of the initial capacitance as the current density increases from 0.5 to 5 mA cm^−2^, while the highest areal capacitance obtained for PC-900/CNTF is 29.1 mF cm^−2^ with the retention of 69.1% at the same condition.

**Figure 7.**
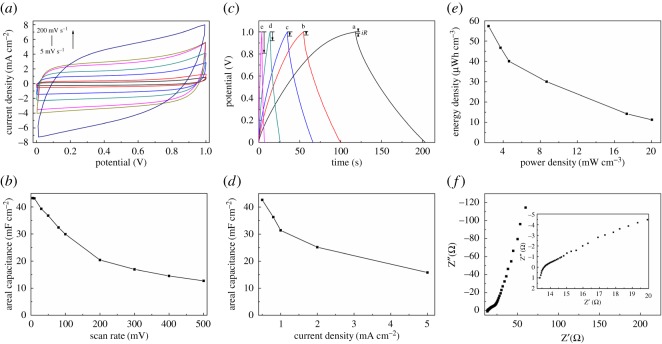
CV curves of the NPC-1-900/CNTF flexible SSC device at different scan rates of 5--200 mV s^−1^ (*a*) (from bottom to top is 5, 10, 30, 50, 80, 100 and 200 mV s^−1^). Relationship of the areal capacitance with different CV scan rates (*b*). GCD curves (*c*) of NPC-1-900/CNTF flexible SSC device at different GCD current densities (a 0.5, b 0.8, c 1.0, d 2.0 and e 5.0 mA cm^−2^). Relationship of the areal capacitance with different current densities (*d*). Ragone plot of the volumetric energy and power density of device (*e*). Nyquist electrochemical impedance spectra of device (*f*) (inset is the EIS in high-frequency region).

In order to further explore the electrochemical property of NPC-1-900/CNTF electrode, a symmetrical flexible SSC was subsequently assembled and tested in two-electrode system, with gel electrolytes as both electrolyte and ion-porous separator for solid-state SCs. The CV curves, GCD and EIS tests were measured at a voltage window (0–1.0 V). As shown in figure 7*a* and electronic supplementary material, figure S6, the CV curves of the NPC-1-900/CNTF device show excellent quasi-rectangular shapes from 5 to 500 mV s^−1^, which indicate that the device has excellent capacitive behaviour. Figure 7*b* shows the plots of the areal capacitance versus CV scan rates for the NPC-1-900/CNTF device. When the scan rate increases from 5 to 500 mV s^−1^, the areal capacitance still keeps about 40% of the initial values. Moreover, the quasi-triangular-shaped GCD curves at different current densities are shown in figure 7*c*. The plot of the areal capacitance versus different current densities for the NPC-1-900/CNTF device is shown in figure 7*d*. The NPC-1-900/CNTF based device can exhibit a high capacitance of 43.2 mF cm^−2^ at 0.5 mA cm^−2^ and still maintain 18 mF cm^−2^ at a high current density of 5 mA cm^−2^, which indicate that the device has fast charge–discharge performance. The areal capacitance value of NPC-1-900/CNTF device calculated from the CV curves is the same as calculated from the GCD curves. Figure 7*e* shows the volumetric power versus volumetric energy Ragone plot, which is used to assess the electrochemical properties of the NPC-1-900/CNTF device. The NPC-1-900/CNTF device exhibits a volumetric energy density of 57.3 µWh cm^−3^ at a power density of 2.4 mW cm^−3^. The Nyquist plots of the device are shown in figure 7*f*; it is obvious from figure 7*f* that the device has small overall internal resistance.

**Figure 8.**
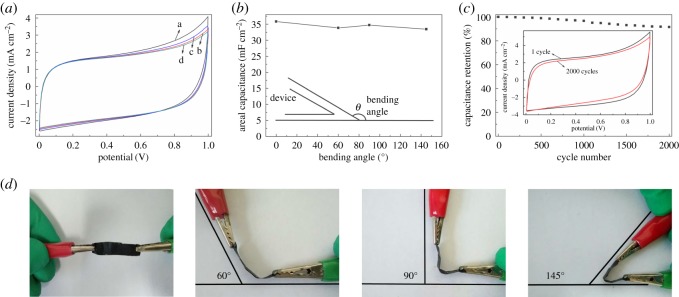
CV curves of NPC-1-900/CNTF flexible SSC device bent with different angles at 50 mV s^−1^ (*a*); areal capacitance of device with different angles (*b*); cycling performance of NPC-1-900/CNTF flexible SSC device measured at 80 mV s^−1^ for 2000 cycles (*c*); optical images of device bent with different angles (*d*).

Nowadays, flexible SSCs have become more and more important because they are flexible, twistable and wearable. To explore the flexible degree of the device, the bending test was conducted under different angles of 0°, 60°, 90° and 145° through CV method at the scan rate of 50 mV s^−1^. The results (figure 8*a*,*b*,*d*) show that the device is highly folded. Another important factor in practical application is the cycling life. As shown in figure 8*c* and electronic supplementary material, figure S7, we can see that the device retains 91.5% and 89.5% of the initial capacitance after 2000 cycles under different angles of 0° and 145°, which indicate that the device possesses an excellent cycling stability and flexibility.

## 4. Conclusion

In summary, we have synthesized a series of NPC from UiO-66 with different ratios of adenine. What is more, the introduction of adenine at different ratios did not change the structure of UiO-66. It was found that when the mole ratio of H_2_BDC and adenine was 9 : 1, the carbonization temperature was 900°C, NPC-1-900 electrode possesses a higher surface area and excellent capacitive performance. The NPC-1-900 electrode with a specific areal capacitance of 128 mF cm^−2^ at 5 mV s^−1^ is superior to the PC-900-CNTF electrode (22.8 mF cm^−2^) with a three-electrode system, indicating that the N-doping improves the capacitive performance of porous carbon. The assembled optimum flexible SSC shows superior areal capacitance 43.2 mF cm^−2^ at the scan rate of 5 mV s^−1^ using PVA/H_3_PO_4_ gel electrolyte. The flexible device shows excellent foldability and cycling stability, which can retain 91.5% of the initial capacitance after 2000 cycles. The present finding provides us a new method to prepare NPC with the use of MOF materials.

## Supplementary Material

Thermogravimetric (TG) analysis;Powder X-ray diffraction ;N2 adsorption/desorption isotherms ; SEM images;CV curves

## Data accessibility

Our data have been deposited in the Dryad Digital Repository (http://dx.doi.org/10.5061/dryad.82600) [47].

## References

[1] Robert CL, Vallé K, Pereira F, Sanchez C 2011 Design and properties of functional hybrid organic-inorganic membranes for fuel cells. Chem. Soc. Rev. 40, 961–1005. (doi:10.1039/c0cs00144a) 21218233 10.1039/c0cs00144a

[2] Choi KM, Jeong HM, Park JH, Zhang YB, Kang JK, Yaghi OM 2014 Supercapacitors of nanocrystalline metal-organic frameworks. ACS Nano 8, 7451–7457. (doi:10.1021/nn5027092) 24999543 10.1021/nn5027092

[3] Simon P, Gogotsi Y 2008 Materials for electrochemical capacitors. Nat. Mater. 7, 845–854. (doi:10.1038/nmat2297) 18956000 10.1038/nmat2297

[4] Zhang LL, Zhao XS 2009 Carbon-based materials as supercapacitor electrodes. Chem. Soc. Rev. 38, 2520–2531. (doi:10.1039/b813846j) 19690733 10.1039/b813846j

[5] Zhai YP, Dou YQ, Zhao DY, Fulvio PF, Mayes RT, Dai S 2011 Carbon materials for chemical capacitive energy storage. Adv. Mater. 23, 4828–4850. (doi:10.1002/adma.201100984) 21953940 10.1002/adma.201100984

[6] Wang L, Feng X, Ren LT, Piao QH, Zhong JQ, Wang YB, Li HW, Chen YF, Wang B 2015 Flexible solid-state supercapacitor based on a metal–organic framework interwoven by electrochemically-deposited PANI. J. Am. Chem. Soc. 137, 4920–4923. (doi:10.1021/jacs.5b01613) 25864960 10.1021/jacs.5b01613

[7] Dong LB, Xu CJ, Li Y, Huang ZH, Kang FY, Yang QH, Zhao X 2016 Flexible electrodes and supercapacitors for wearable energy storage: a review by category. J. Mater. Chem. A 4, 4659–4685. (doi:10.1039/c5ta10582j)

[8] Liu CG, Yu ZN, Neff D, Zhamu A, Jang BZ 2010 Graphene-based supercapacitor with an ultrahigh energy density. Nano Lett. 10, 4863–4868. (doi:10.1021/nll02661q) 21058713 10.1021/nl102661q

[9] Saraf M, Dar RA, Natarajan K, Srivastava AK, Mobin SM 2016 A binder-free hybrid of CuO-microspheres and rGO nanosheets as an alternative material for next generation energy storage application. Chem. Sel. 1, 2826–2833. (doi:10.1002/slct.201600481)

[10] Saraf M, Natarajan K, Mobin SM 2017 Microwave assisted fabrication of a nanostructured reduced graphene oxide (rGO)/Fe_2_O_3_ composite as a promising next generation energy storage material. RSC Adv. 7, 309–317. (doi:10.1039/c6ra24766k)

[11] González A, Goikolea E, Barrena JA, Mysyk R 2016 Review on supercapacitors: technologies and materials. Renew. Sust. Energy Rev. 58, 1189–1206. (doi:10.1016/j.rser.2015.12.249)

[12] Kim C, Lee J, Kim S, Yoon J 2014 TiO_2_ sol–gel spray method for carbon electrode fabrication to enhance desalination efficiency of capacitive deionization. Desalination 342, 70–74. (doi:10.1016/j.desal.2013.07.016)

[13] Chaikittisilp W, Hu M, Wang HJ, Huang HS, Fujita T, Wu KC-W, Chen LC, Yamauchi Y, Ariga K 2012 Nanoporous carbons through direct carbonization of a zeolitic imidazolate framework for supercapacitor electrodes. Chem. Commun. 48, 7259–7261. (doi:10.1039/c2cc33433j) 10.1039/c2cc33433j 22710974

[14] Yang LF, Shi Z, Yang WH 2014 Enhanced capacitive deionization of lead ions using air-plasma treated carbon nanotube electrode. Surf. Coat. Technol. 251, 122–127. (doi:10.1016/j.surfcoat.2014.04.012)

[15] Jia BP, Zou LD 2012 Graphene nanosheets reduced by a multi-step process as high-performance electrode material for capacitive deionisation. Carbon 50, 2315–2321. (doi:10.1016/j.carbon.2012.01.051)

[16] Wang Y, Shi ZQ, Huang Y, Ma YF, Wang CY, Chen MM, Chen YS 2009 Supercapacitor devices based on graphene materials. J. Phys. Chem. C 113, 13 103–13 107. (doi:10.1021/jp902214f)

[17] Wang J et al. 2017 Hierarchical porous carbons with layer-by-layer motif architectures from confined soft-template self-assembly in layered materials. Nat. Commun. 8, 15 717 (doi:10.1038/ncomms15717) 28604671 10.1038/ncomms15717 PMC5472787

[18] Wei HM, Chen HJ, Fu N, Chen J, Lan GX, Qian W, Liu YP, Lin HL, Han S 2017 Excellent electrochemical properties and large CO_2_ capture of nitrogen-doped activated porous carbon synthesised from waste longan shells. Electrochim. Acta 231, 403–411. (doi:10.1016/j.electacta.2017.01.194)

[19] An YF, Yang YY, Hu ZG, Guo BS, Wang XT, Yang X, Zhang QC, Wu HY 2017 High-performance symmetric supercapacitors based on carbon nanosheets framework with graphene hydrogel architecture derived from cellulose acetate. J. Power Sources 337, 45–53. (doi:10.1016/j.jpowsour.2016.10.112)

[20] Guo BS, Yang YY, Hu ZG, An YF, Zhang QC, Yang X, Wang XT, Wu HT, Wu HY 2017 Redox-active organic molecules functionalized nitrogen-doped porous carbon derived from metal-organic framework as electrode materials for supercapacitor. Electrochim. Acta 223, 74–84. (doi:10.1016/j.electacta.2016.12.012)

[21] Frackowiak E 2007 Carbon materials for supercapacitor application. Phys. Chem. Chem. Phys. 9, 1774–1785. (doi:10.1039/b618139m) 17415488 10.1039/b618139m

[22] Yang QH, Xu WH, Tomita A, Kyotani T 2005 The template synthesis of double coaxial carbon nanotubes with nitrogen-doped and boron-doped multiwalls. J. Am. Chem. Soc. 127, 8956–8957. (doi:10.1021/ja052357e) 15969565 10.1021/ja052357e

[23] Zhang FQ, Meng Y, Gu D, Yan Y, Yu CZ, Tu B, Zhao DY 2005 A facile aqueous route to synthesize highly ordered mesoporous polymers and carbon frameworks with Ia3d bicontinuous cubic structure. J. Am. Chem. Soc. 127, 13 508–13 509. (doi:10.1021/ja0545721) 10.1021/ja0545721 16190709

[24] Lu AH, Schüth F 2006 Nanocasting: a versatile strategy for creating nanostructured porous materials. Adv. Mater. 18, 1793–1805. (doi:10.1002/adma.200600148)

[25] Wang L, Han YZ, Feng X, Zhou JW, Qi PF, Wang B 2016 Metal–organic frameworks for energy storage: batteries and supercapacitors. Coord. Chem. Rev. 307, 361–381. (doi:10.1016/j.ccr.2015.09.002)

[26] Zhao Y, Song ZX, Li X, Sun Q, Cheng NC, Lawes S 2016 Metal organic frameworks for energy storage and conversion. Energy Storage Mater. 2, 35–62. (doi:10.1016/j.ensm.2015.11.005)

[27] Yang J, Xiong PX, Zheng C, Qiu HY, Wei MD 2014 Metal-organic frameworks: a new promising class of materials for a high performance supercapacitor electrode. J. Mater. Chem. A 2, 16 640–16 644. (doi:10.1039/c4ta04140b)

[28] Fu DY, Zhou HH, Zhang XM, Han GY, Chang YZ, Li HW 2016 Flexible solid-state supercapacitor of metal-organic framework coated on carbon nanotube film interconnected by electrochemically-codeposited PEDOT-GO. Chem. Sel. 1, 285–289. (doi:10.1002/slct.201600084)

[29] Saraf M, Rajak R, Mobin SM 2016 A fascinating multitasking Cu-MOF/rGO hybrid for high performance supercapacitors and highly sensitive and selective electrochemical nitrite sensors. J. Mater. Chem. A 4, 16 432–16 445. (doi:10.1039/c6ta06470a)

[30] Liu B, Shioyama H, Jiang HL, Zhang XB, Xu Q 2010 Metal–organic framework (MOF) as a template for syntheses of nanoporous carbons as electrode materials for supercapacitor. Carbon 48, 456–463. (doi:10.1016/j.carbon.2009.09.061)

[31] Salunkhe RR, Young C, Tang J, Takei T, Ide Y, Kobayashi N, Yamauchi Y 2016 A high-performance supercapacitor cell based on ZIF-8-derived nanoporous carbon using an organic electrolyte. Chem. Commun. 52, 4764–4767. (doi:10.1039/c6cc00413j) 10.1039/c6cc00413j 26928244

[32] Yuan DS, Chen JX, Tan SX, Xia NN, Liu YL 2009 Worm-like mesoporous carbon synthesized from metal–organic coordination polymers for supercapacitors. Electrochem. Commun. 11, 1191–1194. (doi:10.1016/j.elecom.2009.03.045)

[33] Li B, Shioyama H, Akita T, Xu Q 2008 Metal-organic framework as a template for porous carbon synthesis. J. Am. Chem. Soc. 130, 5390–5391. (doi:10.1021/ja7106146) 18376833 10.1021/ja7106146

[34] Jeon JW et al. 2014 *In situ* one-step synthesis of hierarchical nitrogen-doped porous carbon for high-performance supercapacitors. ACS Appl. Mater. Interfaces 6, 7214–7222. (doi:10.1021/am500339x) 24784542 10.1021/am500339x

[35] Kim J, Young C, Lee J, Heo YU, Park MS, Hossain MSA, Yamauchi Y, Kim JH 2017 Nanoarchitecture of MOF-derived nanoporous functional composites for hybrid supercapacitors. J. Mater. Chem. A 5, 15 065–15 072. (doi:10.1039/c7ta03356g)

[36] Young C, Salunkhe RR, Tang J, Hu CC, Shahabuddin M, Yanmaz E, Hossain MSA, Kim JH, Yamauchi Y 2016 Zeolitic imidazolate framework (ZIF-8) derived nanoporous carbon: the effect of carbonization temperature on the supercapacitor performance in an aqueous electrolyte. Phys. Chem. Chem. Phys. 18, 29 308–29 315. (doi:10.1039/c6cp05555a) 10.1039/c6cp05555a 27731874

[37] Fu DY, Li HW, Zhang XM, Han GY, Zhou HH, Chang YZ 2016 Flexible solid-state supercapacitor fabricated by metal-organic framework/graphene oxide hybrid interconnected with PEDOT. Mater. Chem. Phys. 179, 166–173. (doi:10.1016/j.matchemphys.2016.05.024)

[38] Cavka JH, Jakobsen S, Olsbye U, Guillou N, Lamberti C, Bordiga S, Lillerud KP 2008 A new zirconium inorganic building brick forming metal organic frameworks with exceptional stability. J. Am. Chem. Soc. 130, 13 850–13 851. (doi:10.1021/ja8057953) 10.1021/ja8057953 18817383

[39] Phang WJ, Jo H, Lee WR, Song JH, Yoo K, Kim BS, Hong CS 2015 Superprotonic conductivity of a UiO-66 framework functionalized with sulfonic acid groups by facile postsynthetic oxidation. Angew. Chem. Int. Ed. 54, 5142–5146. (doi:10.1002/ange.201411703) 10.1002/anie.201411703 25726943

[40] Zhang P, Sun F, Xiang Z, Shen Z, Yun J, Cao D 2014 ZIF-derived in situ nitrogen-doped porous carbons as efficient metal-free electrocatalysts for oxygen reduction reaction. Energy Environ. Sci. 7, 442–450. (doi:10.1039/c3ee42799d)

[41] Yan J, Liu JP, Fan ZJ, Wei T, Zhang LJ 2012 High-performance supercapacitor electrodes based on highly corrugated graphene sheets. Carbon 50, 2179–2188. (doi:10.1016/j.carbon.2012.01.028)

[42] Katz MJ, Brown ZJ, Colon YJ, Siu PW, Scheidt KA, Snurr RO, Hupp JT, Farha OK 2013 A facile synthesis of UiO-66, UiO-67 and their derivatives. Chem. Commun. 49, 9449–9451. (doi:10.1039/c3cc46105j) 10.1039/c3cc46105j 24008272

[43] Guo ZH et al. 2013 Nitrogen-doped carbon based on peptides of hair as electrode materials for supercapacitors. Electrochim. Acta 113, 620–627. (doi:10.1016/j.electacta.2013.09.112)

[44] Atchudan R, Edison TNJI, Perumal S, Lee YR 2017 Green synthesis of nitrogen-doped graphitic carbon sheets with use of *Prunus persica* for supercapacitor applications. Appl. Surf. Sci. 393, 276–286. (doi:10.1016/j.apsusc.2016.10.030)

[45] Rana M, Subramani K, Sathish M, Gautam UK 2017 Soya derived heteroatom doped carbon as a promising platform for oxygen reduction, supercapacitor and CO_2_ capture. Carbon 114, 679–689. (doi:10.1016/j.carbon.2016.12.059)

[46] Song K, Ni HF, Fan LZ 2017 Flexible graphene-based composite films for supercapacitors with tunable areal capacitance. Electrochim. Acta 235, 233–241. (doi:10.1016/j.electacta.2017.03.065)

[47] Li H, Fu D, Zhang X-M 2018 Data from: A novel adenine-based metal organic framework derived nitrogen-doped nanoporous carbon for flexible solid-state supercapacitor. Dryad Digital Repository (doi:10.5061/dryad.82600) 10.1098/rsos.171028 PMC5792892 29410815

