# Supplementary material for: A novel adenine-based metal organic framework derived nitrogen-doped nanoporous carbon for flexible solid-state supercapacitor
Source: R Soc Open Sci. 2018 Jan 31;5(1):171028. doi: 10.1098/rsos.171028 (PMC5792892; doi:10.1098/rsos.171028)
Supplement: Thermogravimetric (TG) analysis;Powder X-ray diffraction ;N2 adsorption/desorption isotherms ; SEM images;CV curves [file rsos171028supp1.docx]

## Supporting information for:

## A novel adenine-based MOF-derived nitrogen-doped nanoporous carbon for flexible solid-state supercapacitor

Haowen Li, Dongying Fu^[[1]](#footnote-2)^*, Xian-Ming Zhang*

Institute of Crystalline Materials, Shanxi University, Taiyuan 030006, China


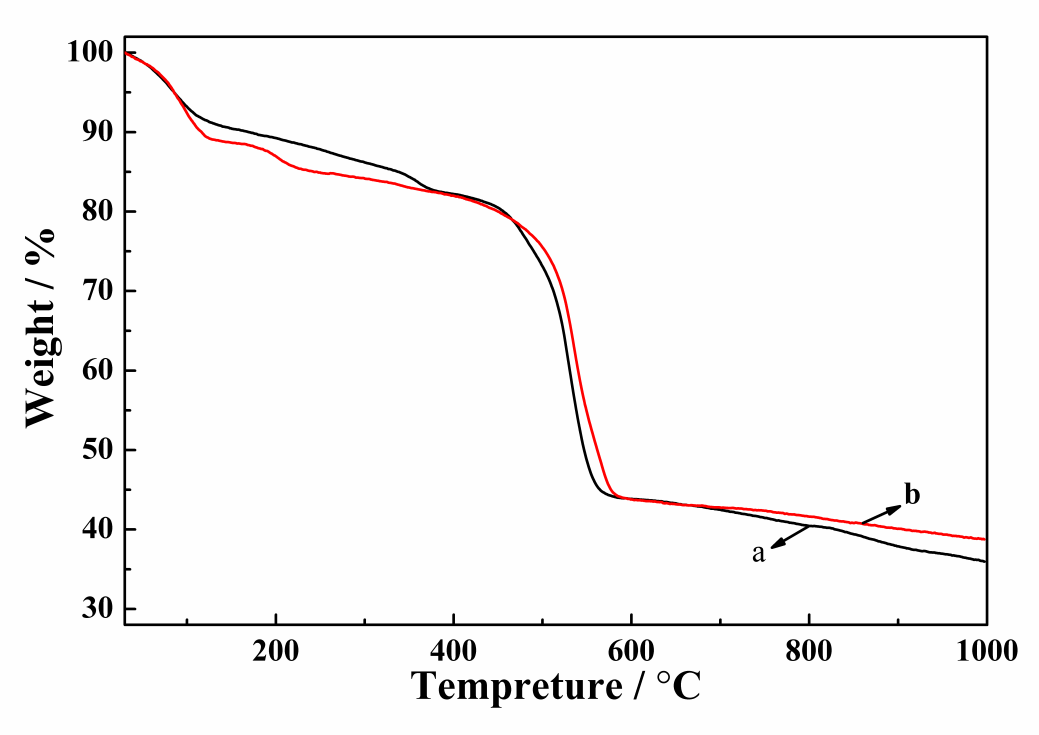


SFig. 1. Thermogravimetric (TG) curve of UiO-66 (a) and N-U-1 (b).


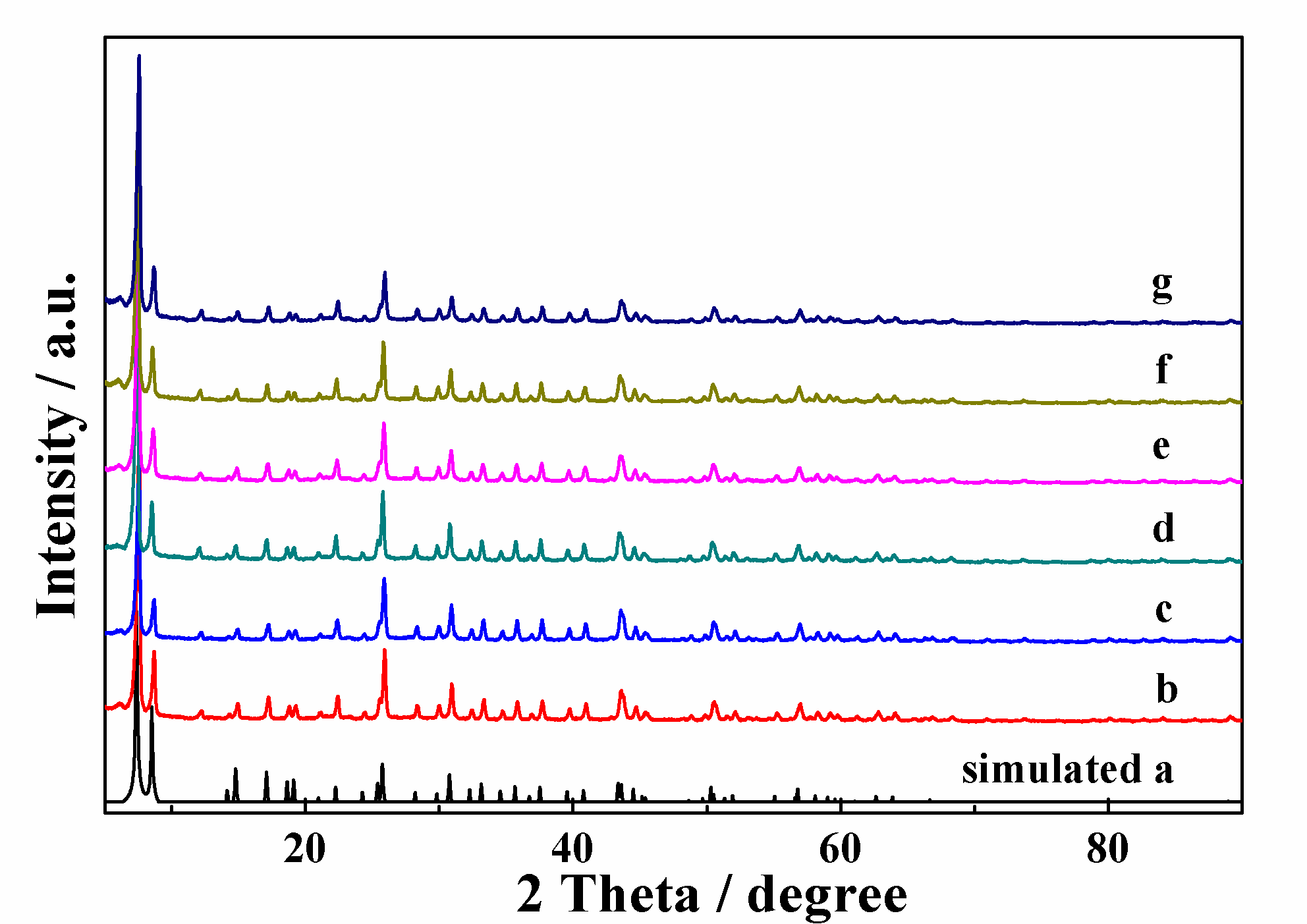


SFig. 2. Powder X-ray diffraction patterns of simulated UiO-66 (a), UiO-66 (b), N-U-1 (c), N-U-2 (d), N-U-3 (e), N-U-4 (f), N-U-5 (g).


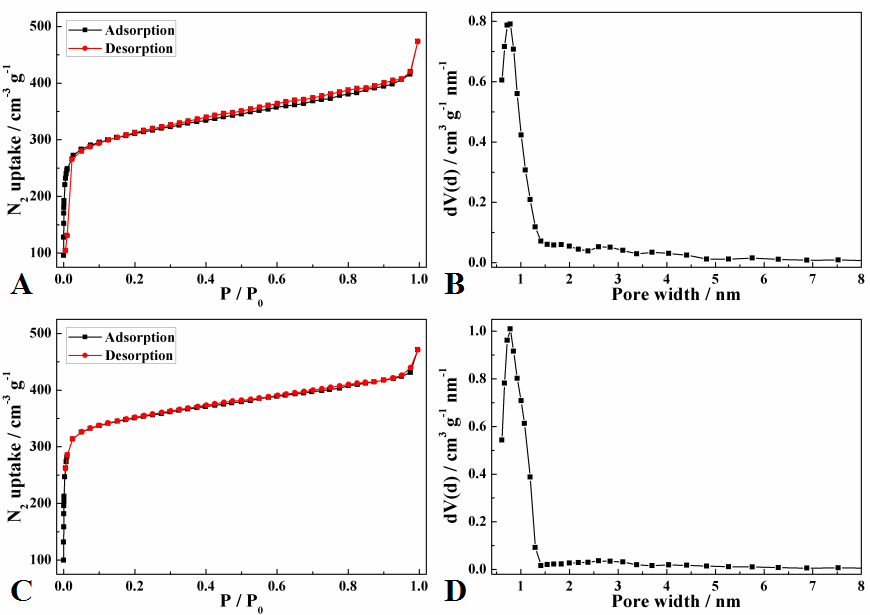


SFig. 3. N_2_ adsorption/desorption isotherms of as-synthesized UiO-66 (A); the pore size distribution of UiO-66 (B); N_2_ adsorption/desorption isotherms of N-U-1 (C); the pore size distribution of N-U-1 (D).


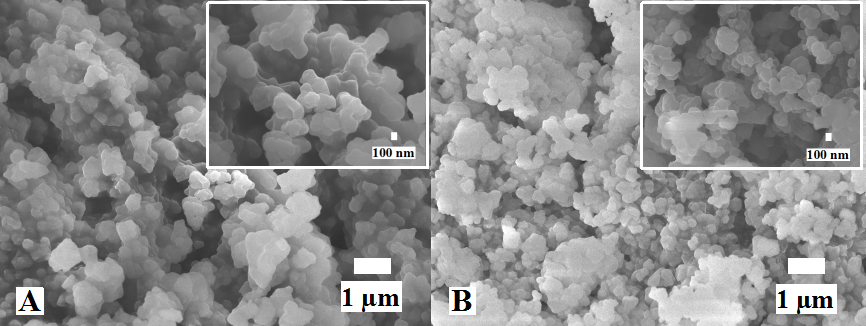


SFig. 4. SEM images of UiO-66 (A) and N-U-1 (B) (The inserted image of A and B is the SEM image at high magnification).


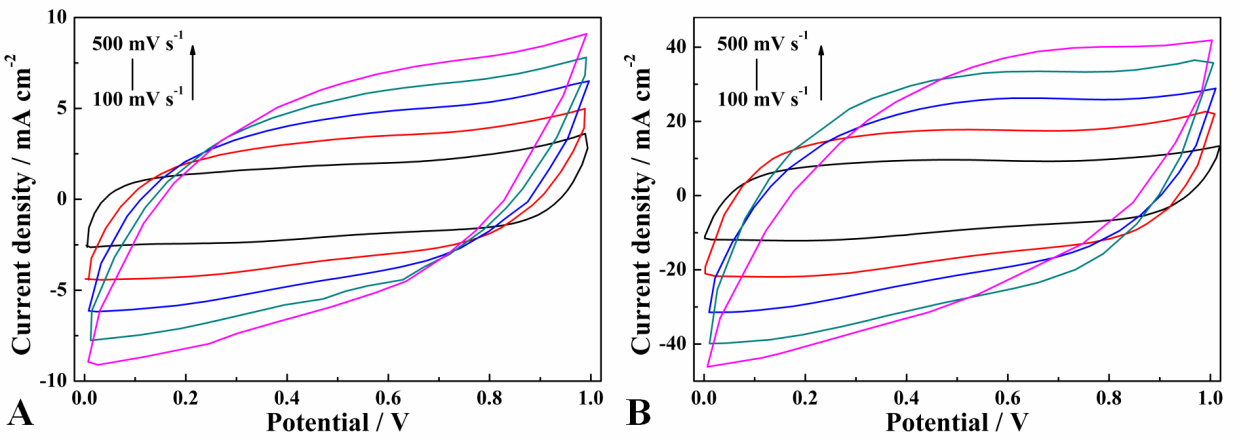


SFig. 5. CV behaviors of PC-900/CNTF (A) and NPC-1-900/CNTF (B) at scan rate of 100-500 mV s^-1^ (from down to up is 100, 200, 300, 400 and 500 mV s^-1^).


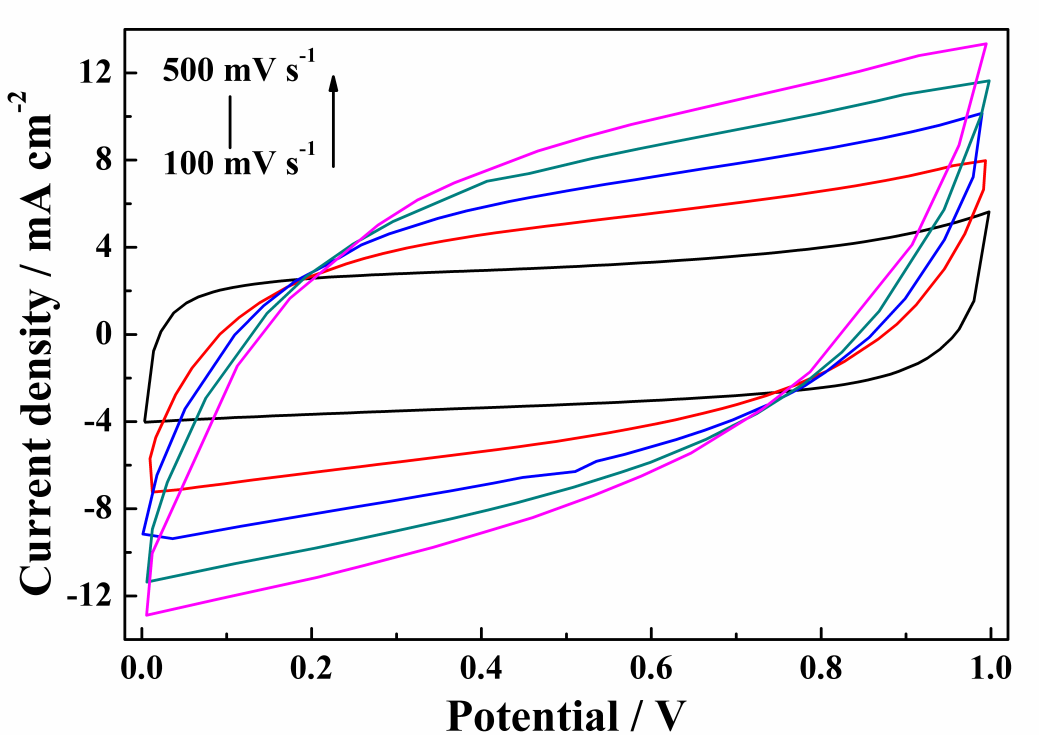


SFig. 6. CV curves of the NPC-1-900/CNTF solid-state SC device at different scan rates range from 100-500 mV s^-1^(from down to up is 100, 200, 300, 400 and 500 mV s^-1^).


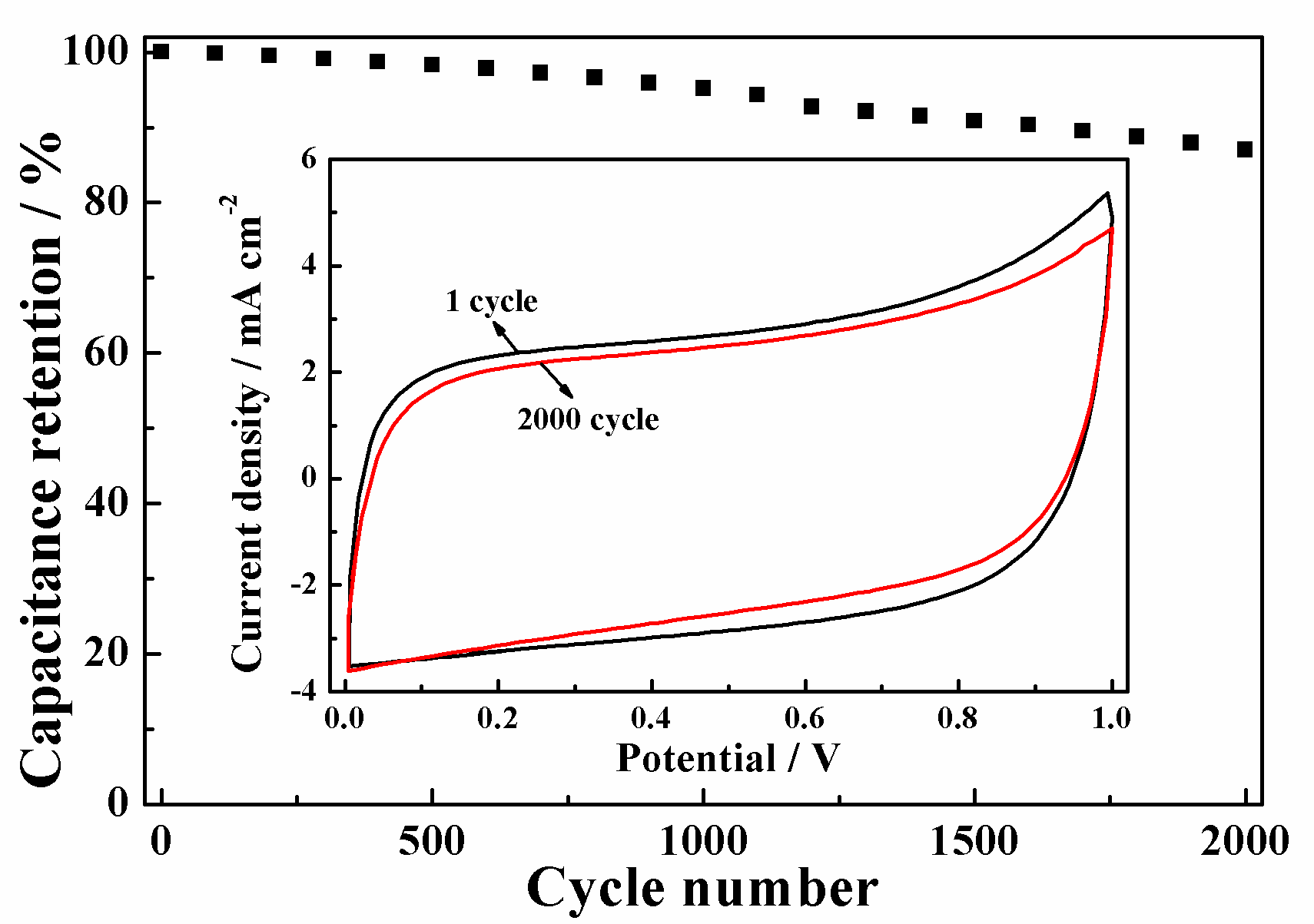


SFig. 7. Cycling performance of NPC-1-900/CNTF flexible SSC device measured at 80 mV s^-1^ for 2000 cycles under bending to 145°.

1. [↑](#footnote-ref-2)
